# Ethanol extract of *Evodia lepta* Merr. ameliorates cognitive impairment through inhibiting NLRP3 inflammasome in scopolamine-treated mice

**DOI:** 10.18632/aging.205486

**Published:** 2024-01-26

**Authors:** Rui-Dan Hu, Wen-Li Zhu, Wei-Yao Lin, Yu-Hui Qiu, Guang-Liang Wu, Xiao-Ying Ding, Zhen-Kun Yang, Qian Feng, Rong-Rong Zhang, Li-Jun Qiao, Ye-Feng Cai, Shi-Jie Zhang

**Affiliations:** 1 State Key Laboratory of Traditional Chinese Medicine Syndrome, The Second Affiliated Hospital of Guangzhou University of Chinese Medicine, Guangzhou 510405, China; 2 Guangdong Provincial Key Laboratory of Translational Cancer Research of Chinese Medicines, Joint International Research Laboratory of Translational Cancer Research of Chinese Medicines, International Institute for Translational Chinese Medicine, School of Pharmaceutical Sciences, Guangzhou University of Chinese Medicine, Guangzhou 510330, China; 3 Department of Neurology, The Second Affiliated Hospital of Guangzhou University of Chinese Medicine, Guangzhou 510000, China; 4 Department of Neurology, Guangdong Provincial Hospital of Chinese Medicine, Guangzhou 510435, China; 5 Department of Gastroenterology, Shenzhen Traditional Chinese Medicine Hospital Affiliated to Nanjing University of Chinese Medicine, Shenzhen, China

**Keywords:** Evodia lepta, dementia, scopolamine, NLRP3 inflammasome

## Abstract

*Evodia lepta* Merr. (Evodia lepta) is a well-known traditional Chinese medicine, which has been widely used in herbal tea. We previously reported that the coumarin compounds from the root of Evodia lepta exhibited neuroprotective effects. However, whether Evodia lepta could inhibit NLRP3 inflammasome in dementia was still unknown. In this study, the components of the Evodia lepta extract were identified by HPLC-Q-TOF HRMS. We employed a scopolamine-treated mouse model. Evodia lepta extract (10 or 20 mg/kg) and donepezil were treated by gavage once a day for 14 consecutive days. Following the behavioral tests, oxidative stress levels were measured. Then, Western blot and immunofluorescence analysis were used to evaluate the expressions of NLRP3 inflammasome. 14 major components of the Evodia lepta extract were identified by HPLC-Q-TOF HRMS. The results of Morris water maze, object recognition task and open field test indicated that Evodia lepta extract could ameliorate cognitive impairment in scopolamine-treated mice. Evodia lepta extract improved cholinergic system. Moreover, Evodia lepta extract improved the expressions of PSD95 and BDNF. Evodia lepta extract suppressed neuronal oxidative stress and apoptosis. In addition, Evodia lepta extract inhibited NLRP3 inflammasome in the hippocampus of scopolamine-treated mice. Evodia lepta extract could protect against cognitive impairment by inhibiting NLRP3 inflammasome in scopolamine-treated mice.

## INTRODUCTION

Dementia is an intelligent impairment syndrome, which is caused by neuronal dysfunction and loss in the brain [1]. However, its mechanism is still unclear. The current medical intervention has not been able to stop the development of dementia, which has brought great burden and pain to the patients, family and society [2].

In dementia, the cognitive impairment is largely caused by the dysfunction of cholinergic neurons and neuronal loss [3]. “Cholinergic hypothesis” believes that dementia is related to the reduction of acetylcholine level and the loss of cholinergic neurons in the brain [4, 5]. Acetylcholine (Ach), a neurotransmitter from the cholinergic neurons, plays an important role in nervous systems. Reduced levels of acetylcholine can cause systemic or local inflammatory responses [6, 7]. In the brain, neuroinflammation clearly occurs in pathologically vulnerable regions, and significantly contributes to dementia pathological processes [8, 9]. NLRP3 (NOD-like receptor thermal protein domain associated protein 3) inflammasome, composed by NLRP3, apoptosis-associated speck-like protein containing a CARD (ASC) and pro-cysteinyl aspartate specific proteinase-1 (pro-Caspase-1), can promote the Aβ-plaque formation, tau pathology, and result in cognitive dysfunction [10, 11]. Therefore, targeted treatments on cholinergic system and NLRP3-related neuroinflammation may be beneficial for dementia symptoms.

*Evodia lepta* Merr. (Evodia lepta) is widely distributed in Guangxi, Guangdong, Hainan, Yunnan and other provinces, which belongs to the rutaceae family. In traditional Chinese medicine, Evodia lepta is often used for lung carbuncle, fever, shortness of breath, sore throat and chickenpox [12]. Many studies have shown that the alkaloids of Evodia lepta have some biological activities, such as inhibiting acetylcholinesterase (AChE), analgesic and anti-tumor [13]. In our previous study, the coumarin compounds extracted from the root of Evodia lepta can inhibit AChE activity in scopolamine-treated SH-SY5Y cells [14]. However, whether ethanol extract of the root of Evodia lepta could inhibit NLRP3 inflammasome remains unknown.

In current study, we employed the scopolamine-induced memory defect model, to study the anti-neuroinflammation effect of ethanol extract of the root of Evodia lepta. Evodia lepta extract could significantly protect against cognitive impairment by inhibiting NLRP3 inflammasome in scopolamine-treated mice.

## MATERIALS AND METHODS

### Materials

Kits for detecting the Malondialdehyde (MDA) level, activity of manganese superoxide dismutase (Mn-SOD), Acetylcholine (Ach) level, activity of Acetylcholinesterase (AChE), and activity of Choline acetyltransferase (ChAT) were purchased from the Nanjing Jiancheng Bioengineering Institute (Nanjing, China). Primary antibodies, Postsynapticdensity 95 (PSD95), BDNF, Cleaved Caspase-1, Bcl-2, Bax and Cleaved Caspase-3, were obtained from Abcam, Inc. (Cambridge, UK). Secondary antibodies (horseradish peroxidase-conjugated anti-rabbit IgG and mouse IgG) and NLRP3 were obtained from Cell Signaling Technology, Inc. (Danvers, MA, USA).

### Drug preparation and analysis

The stem bark of *Evodia lepta* Merr. (5 kg) were extracted by 70% (V/V) ethanol. The solution was concentrated to afford a crude extract (460 g). Finally, 1 g of Evodia lepta extract was determined to contain 10.87 g of crude herb. The LC condition: column, Waters ACQUITY UPLC HSS T3: 1.8 μm, 2.1 mm × 100 mm; column temperature: 45°C; mobile phase A: 100% ultrapure water with 0.1% formic acid; mobile phase B: 100% acetonitrile with 0.1% formic acid; flow rate: 0.4 mL/min. The specific mobile phase changes were as follows: 0–15 min, 10% to 100% B; injection volume, 2 μL.

### HPLC-Q-TOF HRMS analysis

The components analysis was conducted on Agilent 6540 HPLC-MS system. Chromatographic separation was performed on a Waters ACQUITY UPLC HSS T3 column (1.8 μm, 2.1 mm × 100 mm). The mobile phase: (A) was water with 0.1% formic acid, (B) was acetonitrile with 0.1% formic acid. The gradient: 0–8 min: 15–40% B; 8–10 min: 40–60% B; 10–15 min: 60–100% B. The flow rate was 0.4 mL/min. The injection volume was 2 μL. The MS acquisition was performed at positive ionization mode. The ion source parameters: gas temperature 320°C, drying gas 8 L/min, nebulizer 35 psig, sheath gas temperature 350°C, sheath gas flow 11 L/min, voltage 3.5 kV.

### Animals

Male 5-month-old C57BL/6 mice were provided by the Guangdong Province Medicine Experimental Animal Center. They were housed in the Lab of Guangzhou University of Chinese Medicine with constant temperature (21–25°C), a humidity of 50–60%, photoperiod of 12 h, and free access to water and food. All animal experiments were approved by the Guiding Principles for the Care and Use of Laboratory Animals that adopted and promulgated by the United States National Institutes of Health.

### Experimental groups and drug treatment

After acclimatization for 12 weeks, 50 mice were randomly divided into parallel groups including control, scopolamine model, donepezil (5 mg/kg) and Evodia lepta extract (10 or 20 mg/kg), with 10 mice in each group. The mice were treated with Evodia lepta extract and donepezil by gavage once a day for 14 consecutive days. Donepezil was used as a positive drug which is central AChE inhibitor [15]. From the 7th day to the 14th day, all groups were intraperitoneally injected with scopolamine (2 mg/kg) and the control group received the same volume of saline. 30 min later, behavioral test was employed.

### Morris water maze test

The Morris water maze test was performed according to the Morris method [16]. Briefly, mice were subjected to navigation test for 5 consecutive days with four different starting points per day, and the escape latency was recorded. On the 6th day, the mice were subjected to acquisition a probe trial without the platform.

### Object recognition task

The object recognition task was conducted using a previously described protocol [17]. The mice were placed in the testing box and explored freely for 5 min to adapt to two identical objects. On the next day, one of the objects was replaced by a new one, observing the trajectory of mice and the time to explore the two objects.

### Open field test

The mice were placed alone in the center of a square arena (Med Associates Inc, St. Albans, VT, USA, 40 cm × 40 cm) and allowed to move freely for 5 min. Experimental instrument recorded the changes of the mice. After each experiment, the arena was disinfected with 75% alcohol.

### Western blot analysis

The brain tissues were weighed and homogenized on ice in RIPA Lysis Buffer, and centrifuged at 12, 000 × *g* for 10 min at 4°C. Then the lysate was boiled with loading buffer at 100°C for 10 min. The protein samples were separated by SDS-PAGE analysis gel and transferred onto polyvinylidene difluoride (PVDF) membranes. After being blocked with 5% skimmed milk for 70 min, the membranes were incubated overnight with the primary antibodies. Then, the membranes were washed and incubated with secondary antibody for 1 h. The Western blot method was used to detect the expression level of PSD-95, BDNF, Bcl-2, BAX, Pro Caspase-3, Cleaved Caspase-3, NLRP3, and Cleaved Caspase-1. Bands were detected by using an ECL chemiluminescent kit and quantified using NIH ImageJ software.

### Measurement of MDA, SOD, ACh, ChAT and AChE

The brain tissues were homogenized and collected supernatants by centrifugation for biochemical assays. According to the kit instructions, we used the supernatants to detect the MDA level, the SOD activity; the ACh level, the ChAT and AChE activities.

### Immunohistochemistry

The brain tissue sections were first baked in an oven at 60°C for 1 h followed by deparaffinization and rehydration. The sections were incubated in 3% H_2_O_2_ at 37°C for 10 min. After washed by PBS, the sections were treated with sodium citrate buffer 1 × at high temperature performed antigen retrieval. Then, the sections were blocked with 5% BSA for 1 h, incubated by the primary antibody overnight at 4°C, and followed by a secondary antibody incubation. Subsequently, the sections were mounted with anti-fluorescence quenching sealing liquid (including DAPI).

### Statistical analysis

Data analysis was used by SPSS 19.0 and GraphPad Prism 5 software. Statistical significance was analyzed using one-way analysis of variance (ANOVA) followed by Dunn’s test. The level of statistical significance for all tests was *P* < 0.05, *P* < 0.01.

### Data availability statement

All data or resources used in the paper are available by reasonable requirements to the correspondence authors.

## RESULTS

### Identification of the major components of Evodia lepta extract by HPLC-Q-TOF HRMS

**Figure 1:**
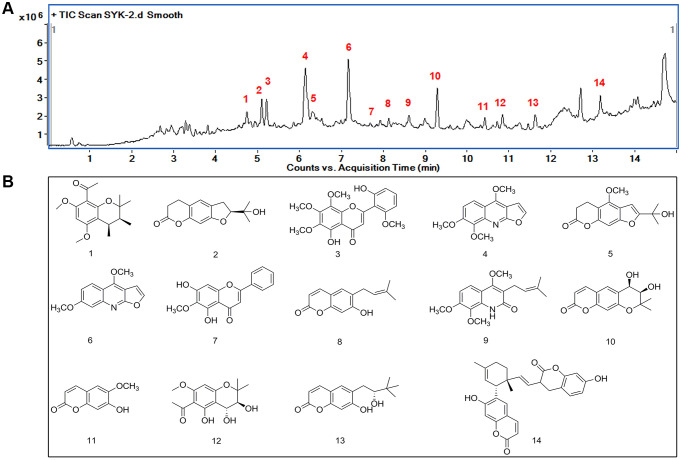
**Identification of major components of Evodia lepta extract by HPLC-Q-TOF HRMS.** (**A**) TIC spectrometry of the EtOH extract of EL, 14 peaks were identified as the characteristic compounds of the EtOH extract of EL. (**B**) Chemical structure of the 14 compounds.

Under optimized chromatographic conditions, 14 components in the TIC chromatograms of the ethanol extract of Evodia lepta sample were identified and assigned by comparing the m/z with those of the reference compounds (Figure 1A, 1B). The 14 major compounds in the ethanol extract of Evodia lepta were tentatively assigned (Table 1).

**Table 1:** The names and formula of the 14 compounds.

**Number**	**Name**	**Formula**
1.	1-((3*S*,4*S*)-3,4-dihydroxy-5,7-dimethoxy-2,2-dimethylchroman-8-yl) ethanone	C_17_H_24_O_4_
2.	(−)-nodakenetin	C_14_H_16_O_4_
3.	Neobaicalein	C_19_H_18_O_8_
4.	skimmianine	C_14_H_13_NO_4_
5.	(+)-peucedanol	C_15_H_16_O_5_
6.	2-(1-Hydroxy-1-methylethyl)-4-methoxy-7H-furo[3,2-g](1) benzopyran-7-one	C_13_H_11_NO_3_
7.	Evolitrin	C_16_H_12_O_5_
8.	Oroxylin A	C_14_H_14_O_3_
9.	Demethylsuberosin	C_17_H_21_NO_4_
10.	Preskimmianine	C_14_H_14_O_5_
11.	*cis*-Decursidinol	C_10_H_8_O_4_
12.	Scopoletin	C_14_H_18_O_6_
13.	Leptin A	C_15_H_18_O_4_
14	(+)-peucedanol	C_28_H_26_O_6_

### Evodia lepta extract improves cognitive impairment and anxiety in scopolamine-treated mice

**Figure 2:**
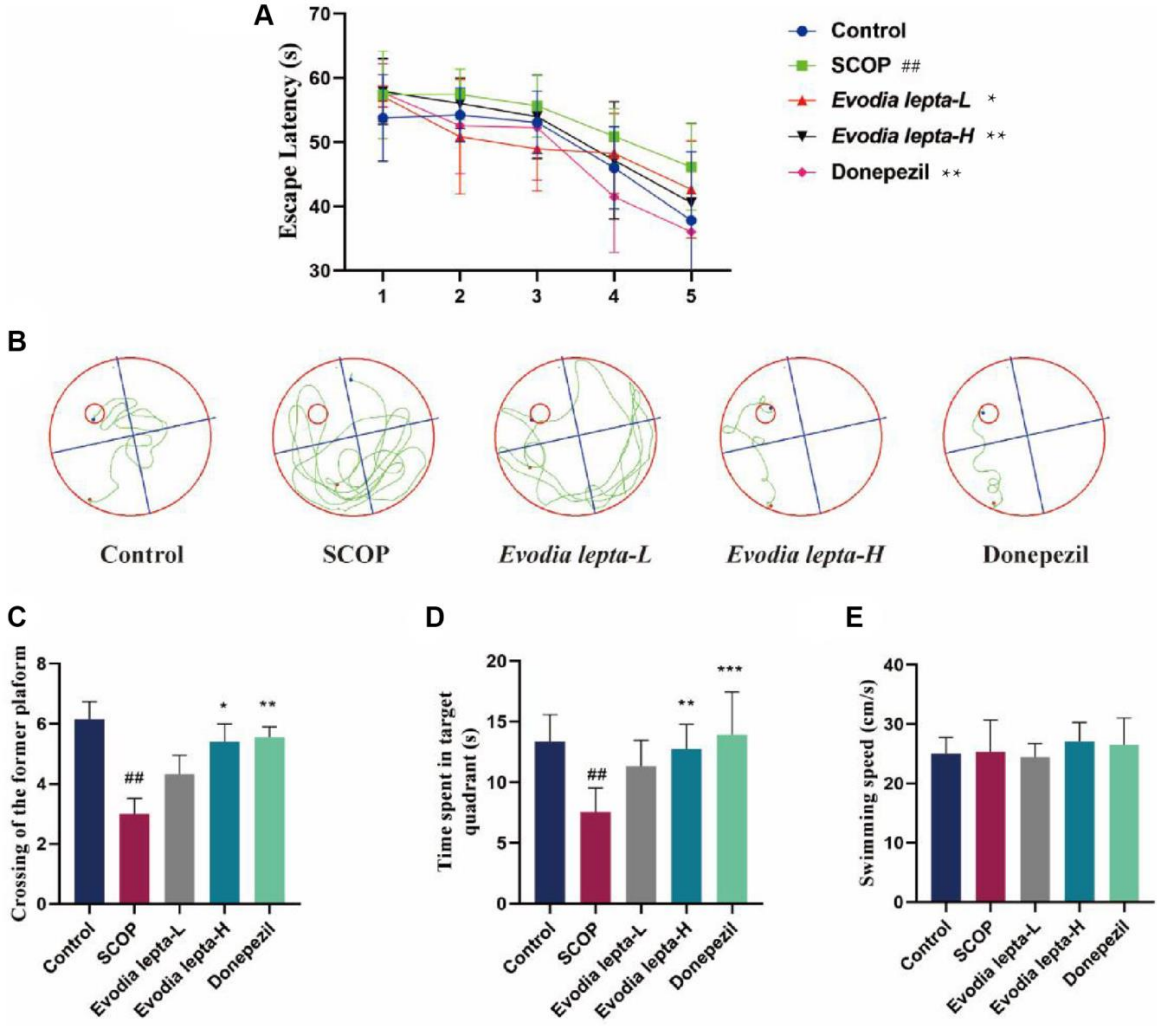
**Evodia lepta extract improves cognitive impairment (Morris Water Maze) in scopolamine-treated mice.** (**A**) Escape latency measured as mean time (s) during the navigation test. (**B**) Representative swim traces of each group. (**C**) Times of crossing the target platform in the probe trial. (**D**) Time spent in the target quadrant in the probe trial. (**E**) The swimming speed in the probe trial. Evodia lepta 10 (10 mg/kg/d); Evodia lepta 20 (20 mg/kg/d). Data represent mean ± SD (*n* = 10 per group). ^##^*P* < 0.01, vs. Control; ^*^P < 0.05, ^**^P < 0.01, ^***^P < 0.001, vs. SCOP.

**Figure 3:**
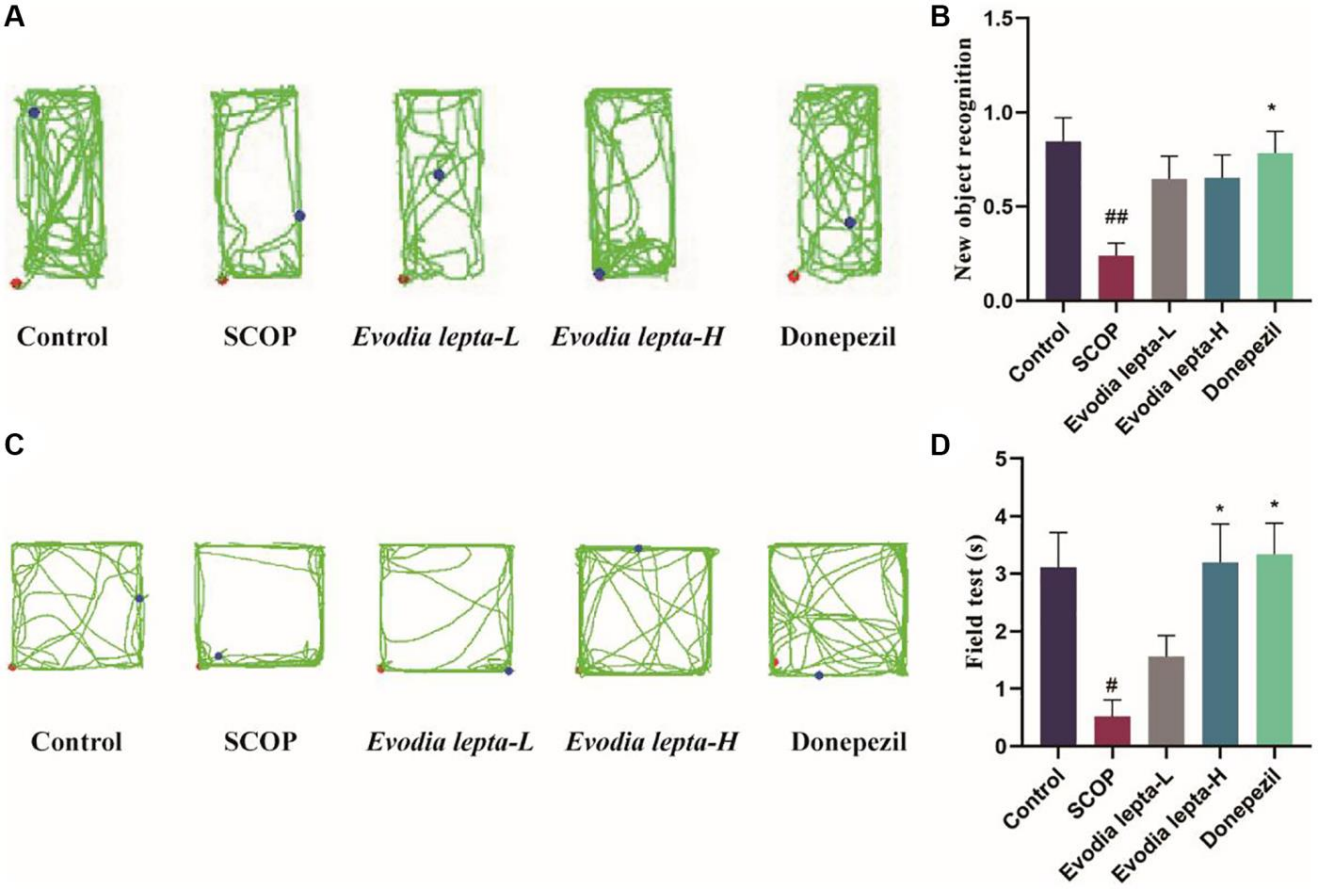
**The effect of Evodia lepta on the working memory impairment and anxiety (Object recognition task and Open field test) induced by scopolamine.** (**A**) Representative trajectory for each group in the new object recognition. (**B**) Mean time (±SEM) spent by each respective group exploring a reference object and a new object. (**C**) Representative trajectory for each group in the open field test. (**D**) Center exploration time. Evodia lepta 10 (10 mg/kg/d); Evodia lepta 20 (20 mg/kg/d). Data represent mean ± SD (*n* = 10 per group). ^#^*P* < 0.05, ^##^*P* < 0.01, vs. Control; ^*^P < 0.05, vs. SCOP.

Firstly, we used Morris Water Maze test to access the effect of Evodia lepta extract on memory protection. The escape latency of the mice gradually decreased during the five consecutive days. The scopolamine group required more time to find the hidden platform when compared with the control group. The Evodia lepta administered groups and the positive group significantly improved the situation (Figure 2A). The representative swimming trails for the five groups showed the similar trends (Figure 2B). In the probe test, the high-dose Evodia lepta group improved the crossing times of the targeting platform (Figure 2C) and the time spent in the target quadrant (Figure 2D). The swimming speed was showed at no change (Figure 2E). In the novel recognition test, the scopolamine group was significantly lower than control group. Compared with scopolamine group, Evodia lepta groups were higher, but the effect is not significant (Figure 3A, 3B). In the open field test, the time of exploring inner squares (Figure 3C, 3D) were better than that in the scopolamine group. These results suggested that Evodia lepta extract could improve the cognitive impairment and anxiety in scopolamine-treated mice.

### Evodia lepta extract ameliorates cholinergic system deficiency in scopolamine-treated mice

**Figure 4:**
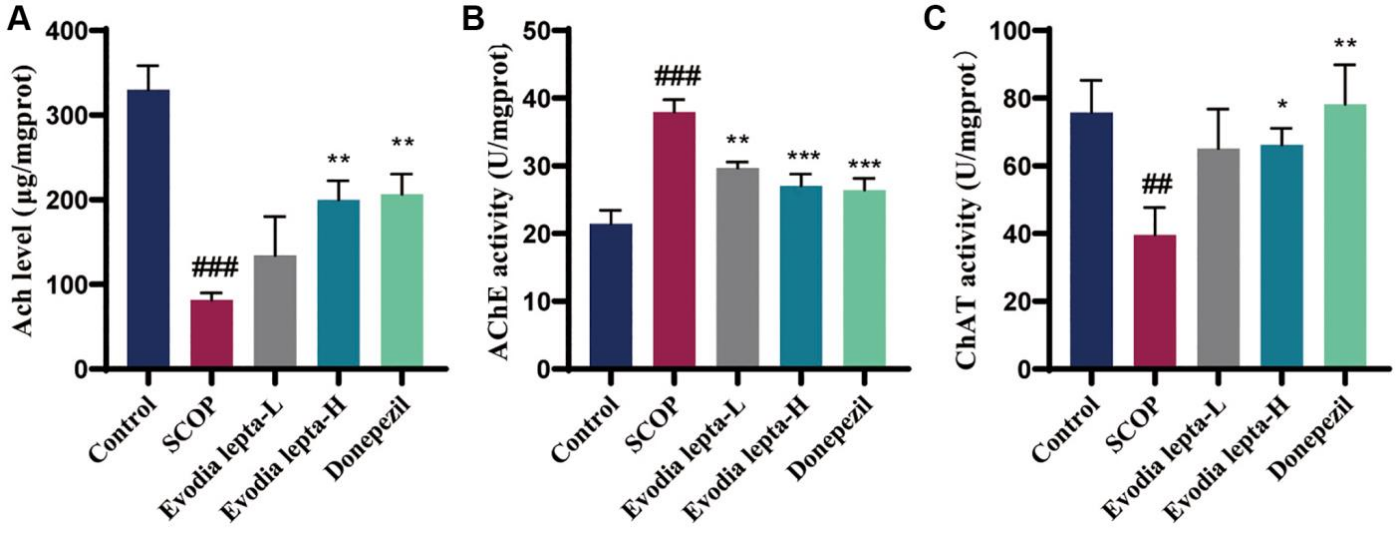
**Evodia lepta extract ameliorates cholinergic system deficiency in scopolamine-treated mice.** (**A**–**C**) The level of Ach and activities of AChE and ChAT. Evodia lepta 10 (10 mg/kg/d); Evodia lepta 20 (20 mg/kg/d). Data represent mean ± SD (*n* = 6 per group). ^##^*P* < 0.01, ^###^*P* < 0.001, vs. Control; ^*^P < 0.05, ^**^P < 0.01, ^***^P < 0.001, vs. SCOP.

As shown in Figure 4A–4C, we evaluated the effects of Evodia lepta extract on the cholinergic system deficiency. Scopolamine treatment significantly reduced Ach level and ChAT activity, and increased AChE activity. Evodia lepta extract and donepezil significantly reversed the changes. These results suggested that Evodia lepta extract could ameliorate cholinergic system deficiency in scopolamine-treated mice.

### Evodia lepta extract protects the synaptic and neurotrophic factors in scopolamine-treated mice

**Figure 5:**
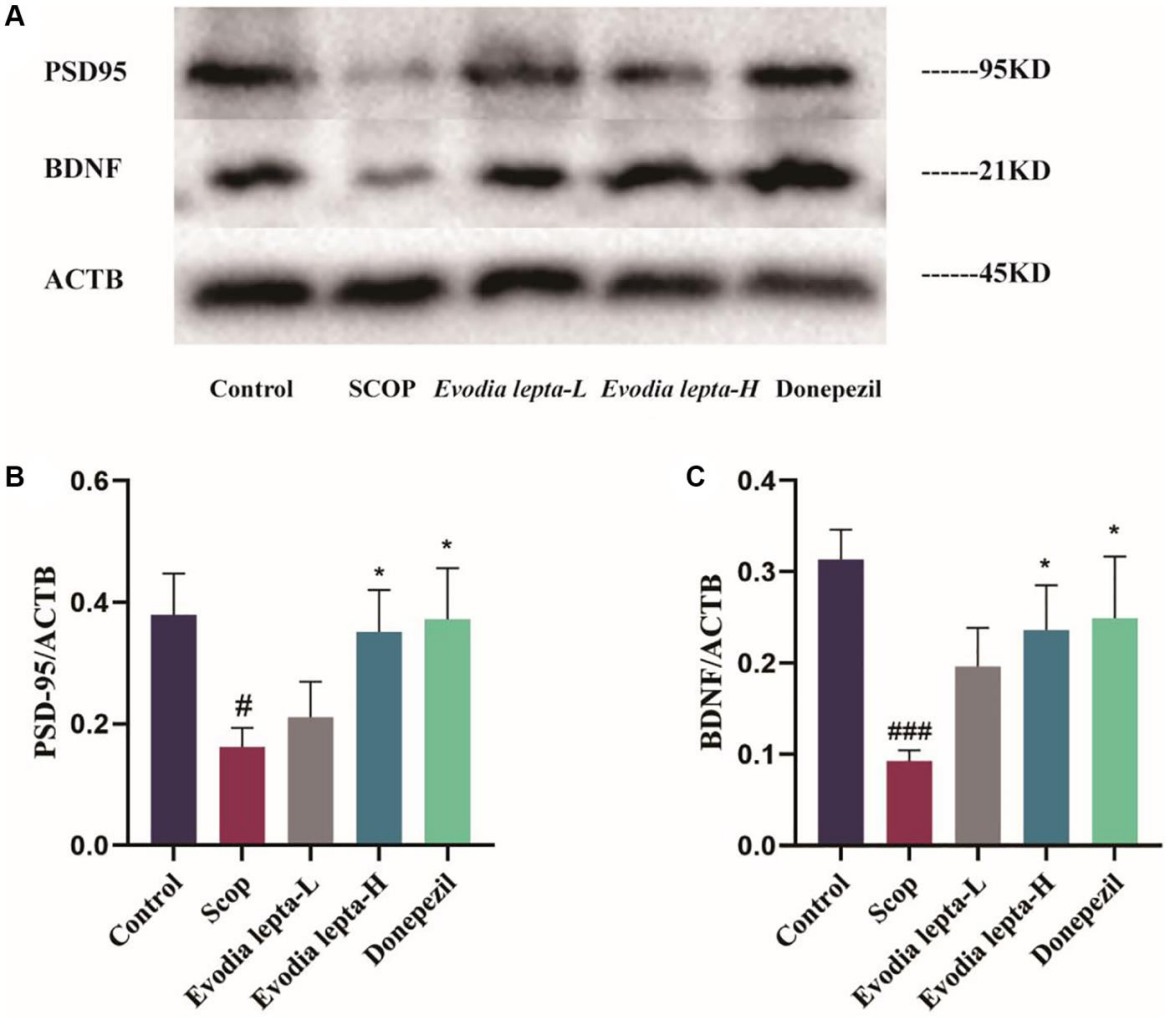
**Evodia lepta extract protects the synaptic and neurotrophic factors in scopolamine-treated mice.** (**A**) Western blot of PSD95 and BDNF. (**B**, **C**) The expressions of PSD95 and BDNF. Evodia lepta 10 (10 mg/kg/d); Evodia lepta 20 (20 mg/kg/d). Data represent mean ± SD (*n* = 3 per group). ^#^*P* < 0.05, ^##^*P* < 0.01, vs. Control; ^*^P < 0.05, vs. SCOP.

As shown in Figure 5A–5C, the levels of PSD95 and BDNF were decreased in the group of scopolamine group. After Evodia lepta extract and donepezil treatment, PSD95 and BDNF levels were increased, but there was no significant difference in PSD95 between the treatment group and scopolamine group. These results indicated that Evodia lepta extract could improve scopolamine-induced neurodegeneration in mice.

### Evodia lepta extract inhibits oxidative stress in scopolamine-treated mice

**Figure 6:**
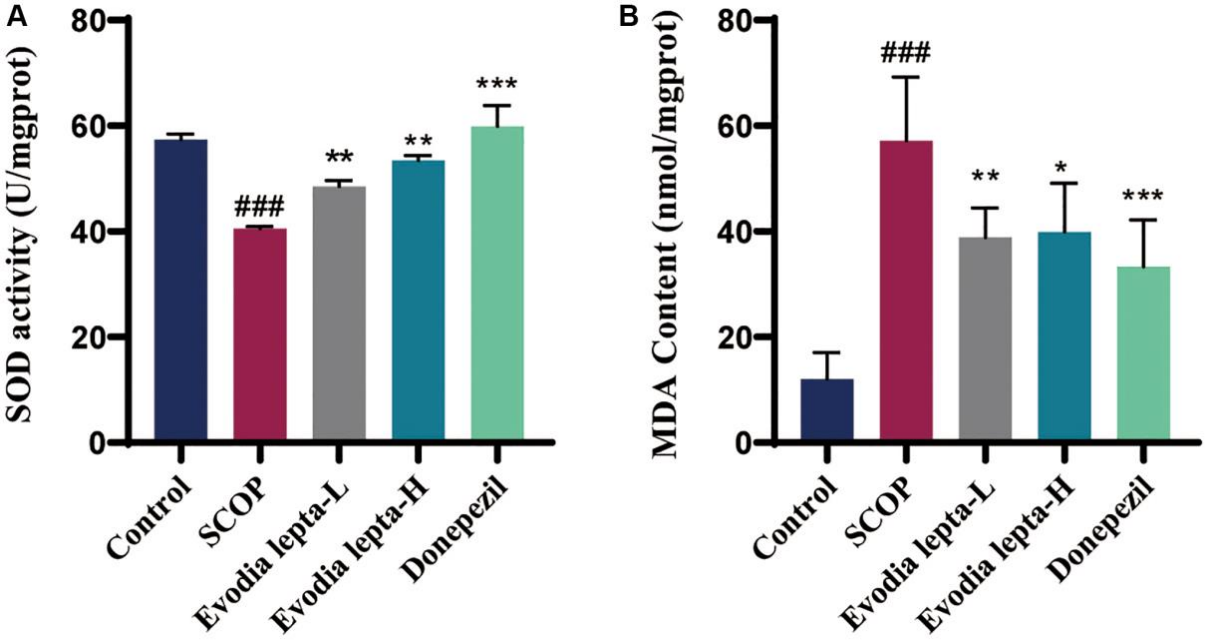
**Evodia lepta extract inhibits oxidative stress in scopolamine-treated mice.** (**A**, **B**) The level of MDA and SOD. Evodia lepta 10 (10 mg/kg/d); Evodia lepta 20 (20 mg/kg/d). Data represent mean ± SD (*n* = 6 per group). ^###^*P* < 0.001, vs. Control; ^*^P < 0.05, ^**^P < 0.01, ^***^P < 0.001, vs. SCOP.

We also evaluated the effect of Evodia lepta extract on oxidative stress. Evodia lepta and donepezil decreased the level of MDA and increased the activity of SOD, compared to the scopolamine group (Figure 6A, 6B). These results indicated that Evodia lepta extract significantly improved oxidative stress.

### Evodia lepta extract ameliorates apoptosis in scopolamine-induced mice

**Figure 7:**
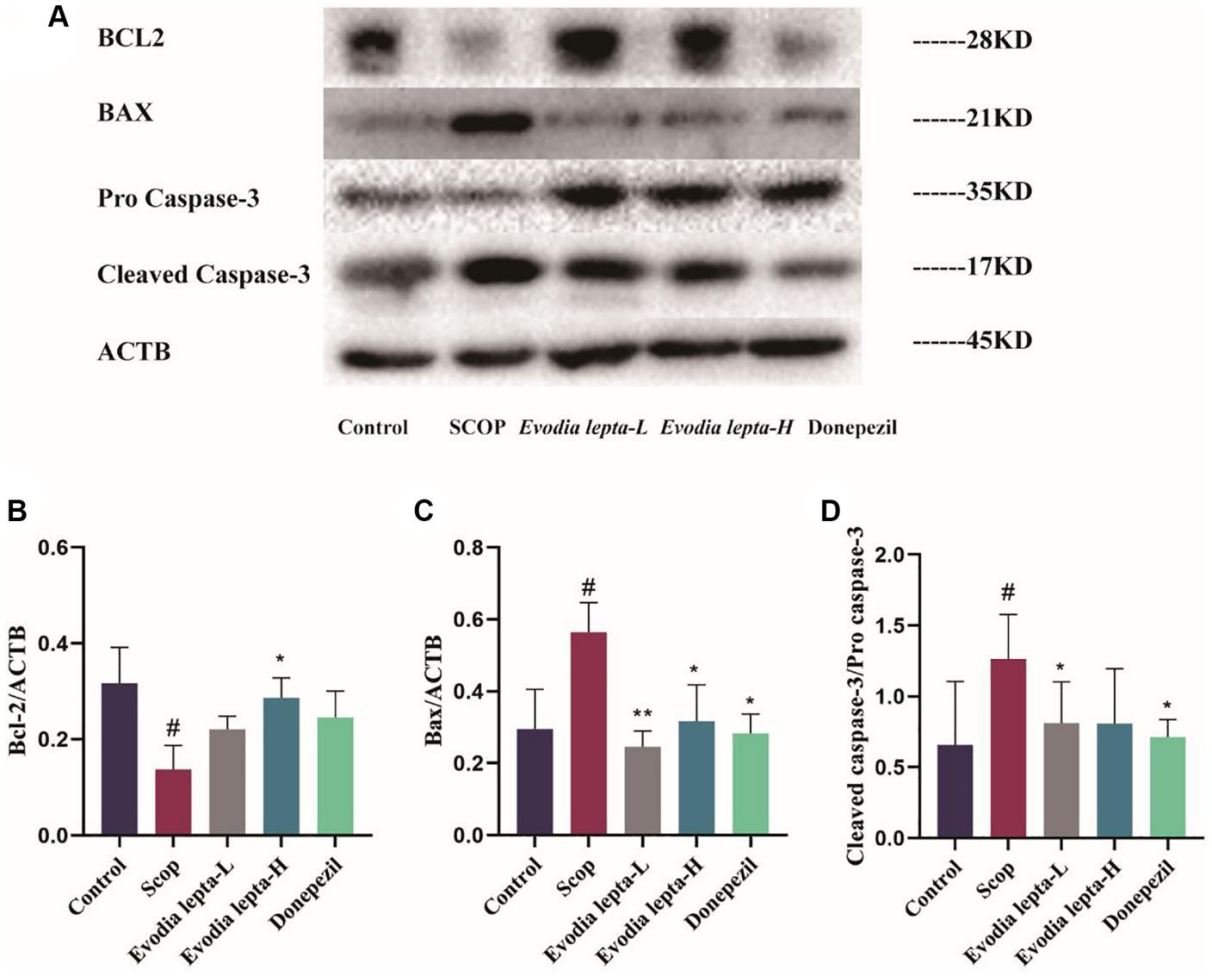
**Evodia lepta extract ameliorates apoptosis in scopolamine-induced mice.** (**A**) Western blot of BCL-2, Bax and Cleaved Caspase-3. (**B**–**D**) The expressions of BCL-2, Bax and Cleaved Caspase-3. Evodia lepta 10 (10 mg/kg/d); Evodia lepta 20 (20 mg/kg/d). Data represent mean ± SD (*n* = 3 per group). ^#^*P* < 0.05, vs. Control; ^*^P < 0.05, ^**^P < 0.01, vs. SCOP.

We elucidated the effect of Evodia lepta extract on neuronal apoptosis by detecting the expressions of apoptotic proteins. The level of BAX and Cleaved Caspase-3 were increased and Bcl-2 was decreased in the scopolamine-induced mice, Evodia lepta extract and donepezil significantly improved the situation (Figure 7A–7D). These results indicated that Evodia lepta extract ameliorated apoptosis in scopolamine-induced mice.

### Evodia lepta extract inhibits NLRP3 inflammasome in scopolamine-treated mice

**Figure 8:**
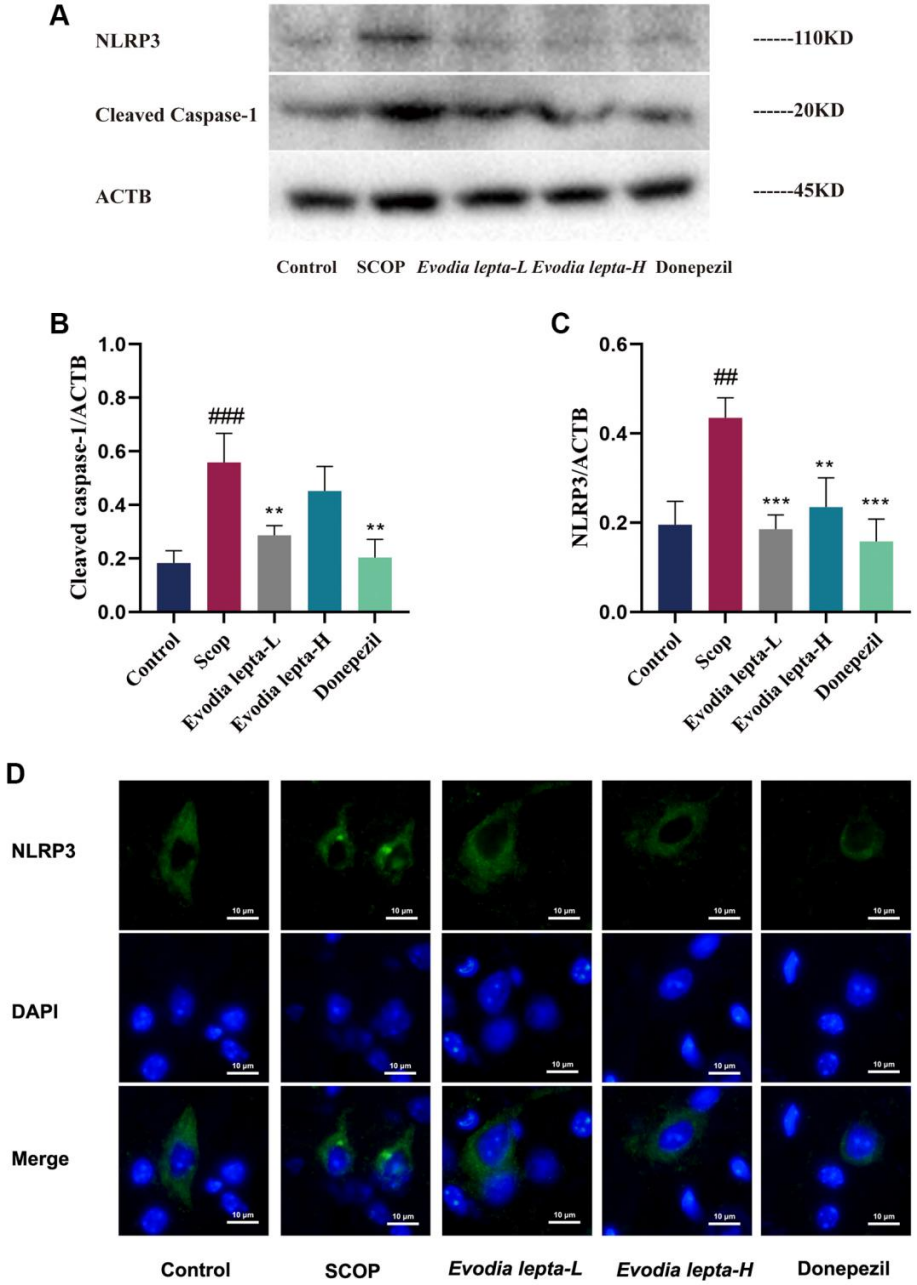
**Evodia lepta extract inhibits NLRP3 inflammasome in scopolamine-treated mice.** (**A**) Western blot of NLRP3 and Cleaved Caspase-1. (**B**, **C**) The expressions of NLRP3 and Cleaved Caspase-1. (**D**) Subcellular localization of NLRP3 was observed by immunofluorescence. Evodia lepta 10 (10 mg/kg/d); Evodia lepta 20 (20 mg/kg/d). Data represent mean ± SD (*n* = 3 per group). ^#^*P* < 0.01, ^##^*P* < 0.001, vs. Control; ^*^P < 0.01, ^**^P < 0.001, vs. SCOP.

To investigate the effect of Evodia lepta extract on NLRP3 inflammasome, we measured it with immunofluorescence and Western blot. As shown in Figure 8, Western blot result demonstrated that the expressions of NLRP3 and Cleaved Caspase-1 were significantly increased in scopolamine-treated mice. Evodia lepta extract and donepezil significantly decreased the expressions of NLRP3 and Cleaved Caspase-1 (Figure 8A–8C). Consistently, the immunofluorescence result showed the similar trends (Figure 8D). These results indicated that Evodia lepta extract could inhibit NLRP3 inflammasome in scopolamine-induced mice.

## DISCUSSION

Previous reports have shown that abnormal central cholinergic system is closely related to the pathogenesis of dementia, and Evodia lepta acts on the cholinergic system by inhibiting AChE activity [18]. Previously, we also found the coumarin compounds from the root of Evodia lepta had the neuroprotective effects by inhibiting AChE activity in scopolamine-treated SH-SY5Y cells [14]. Based on the above studies, we studied the mechanism of Evodia lepta in dementia. In this study, a cognitive impairment model caused by scopolamine was established, which exhibited cholinergic neuronal dysfunction and memory damage [19]. In addition, anxiety is a common symptom of dementia [20, 21]. Anxiety is a major mental disorder in later life of adults living with dementia [22, 23]. Since many dementia patients show anxiety-like behavior at the early stage of the disease, we also conducted Morris Water Maze test, novel recognition test and open field test. These results confirmed that the EtOH extract of Evodia lepta could protect the learning and memory impairment and anxiety behavior induced by scopolamine in mice.

The cholinergic neuron theory is considered to be one of the core of the pathogenesis of dementia [24]. Ach is released by presynaptic neurons and widely exists in the brain. Its main function is to maintain postsynaptic membrane excitability and neural signal transmission, which plays an important role in learning and memory. AChE and ChAT are the key enzymes in biological nerve conduction. ChAT is involved in the production of Ach, and AChE is involved in the hydrolysis and cleavage of Ach, which consumed the levels of Ach [5, 25]. It was found that the local concentration of AChE around the amyloid deposition area and tangles increase the lesions development, which also promote the production and accumulation of Aβ [26]. Hence, in this study, in order to clarify the effect of Evodia lepta extract on cholinergic system, AChE activity, ChAT activity and Ach level in the hippocampus of scopolamine-induced mice were examined. The results confirmed that Evodia lepta extract could regulate AChE activity, ChAT activity and Ach level in scopolamine-treated mice.

The cholinergic nervous system can influence synaptic transmission and synaptic plasticity [27]. Synaptic plasticity is important for brain learning and memory. Some evidences suggested that synaptic plasticity damages were increased in dementia [28]. BDNF, an important neurotrophic factor in neurons and glial cells, can regulate synaptic genesis and synaptic plasticity [29]. It is associated with several signaling pathways in synapse formation, including upregulating PSD-95, an important scaffolding protein in excitatory synapses [30, 31]. Therefore, BDNF and PSD-95 can affect memory function by promoting neuronal survival and differentiation. In this study, we found that Evodia lepta extract increased the levels of BDNF and PSD95 in scopolamine-induced mice.

Memory deficits induced by the cholinergic nervous system are often accompanied by oxidative stress [32, 33], which induces ROS, and leads to neuronal apoptosis in neurodegenerative diseases. SOD provides a major defense against oxidative stress by scavenging free radicals. MDA is a lipid index of each oxidation, indicating the overproduction of reactive oxygen species [34, 35]. In our study, Evodia lepta extract decreased the level of MDA and increased the activity of SOD in scopolamine-treated mice.

NLRP3, an inflammasome receptor, generates a chronic inflammatory environment and regulate the maturation of its downstream target Caspase-1, followed by apoptosis and cytokine release, leading to the development and progression of dementia [36, 37]. NLRP3 inflammasome is a trigger for the pathogenesis of AD. NLRP3 activation produces IL-1β, IL-18 and other cytokines, and then promotes Aβ-plaque formation, resulting in cognitive dysfunction. Apoptosis plays an important role in dementia neuron loss, and Caspases and Bcl-2 protein families are central components of apoptotic. In our study, Evodia lepta extract inhibited NLRP3 inflammasome (decreased the level of NLRP3 and Cleaved caspase-1) and pro-apoptosis protein (BAX and caspase-3), and increased anti-apoptosis protein (Bcl-2) in scopolamine-induced mice.

## CONCLUSIONS

These results suggested that Evodia lepta extract might prevent against memory loss by inhibiting NLRP3 inflammasome. NLRP3 inflammasome may be an ideal target for preventing against cognitive decline and neurodegeneration. Further studies are still needed to identify the active compound in Evodia lepta extract which targeting NLRP3 inflammasome.
